# Metabolic regulation of lifespan from a *C. elegans* perspective

**DOI:** 10.1186/s12263-019-0650-x

**Published:** 2019-08-15

**Authors:** Kathrine B. Dall, Nils J. Færgeman

**Affiliations:** Department of Biochemistry and Molecular Biology, Villum Center for Bioanalytical Sciences, University of Southern Denmark, Campusvej 55, 5230 Odense M, Denmark

**Keywords:** *Caenorhabditis elegans*, Aging, Longevity, Dietary restriction, Autophagy, HLH-30/TFEB, Metabolism, Epigenetics

## Abstract

Decline of cellular functions especially cognitive is a major deficit that arises with age in humans. Harnessing the strengths of small and genetic tractable model systems has revealed key conserved regulatory biochemical and signaling pathways that control aging. Here, we review some of the key signaling and biochemical pathways that coordinate aging processes with special emphasis on *Caenorhabditis elegans* as a model system and discuss how nutrients and metabolites can regulate lifespan by coordinating signaling and epigenetic programs. We focus on central nutrient-sensing pathways such as mTOR and insulin/insulin-like growth factor signaling and key transcription factors including the conserved basic helix-loop-helix transcription factor HLH-30/TFEB.

## Background

By establishing *Caenorhabditis elegans* (*C. elegan*s) as a genetic model organism a little more than 50 years ago, Brenner [1] opened the door to the possibility of uncovering central molecular mechanisms governing cellular connectivity and longevity. Not only was *C. elegans* the first eukaryotic multicellular organism to have its complete genetic profile sequenced [2], the cell linage of each individual cell in the worm has been mapped [3–5], and each cell has been characterized by electron microscopy. In the laboratory, the transparent nematode has a lifespan of approximately 3 weeks, and its rapid development allows it to progress from egg, through four larval stages, and to a fertile adult in only 3 days at 20 °C. These properties have established *C. elegans* as a highly tractable and applied model in longevity studies. Besides the short cultivation period, the feeding habit of *C. elegans* has made it an excellent system for genetic manipulation as RNAi can be performed by feeding the animals *E. coli* expressing a specific dsRNA, targeting a specific mRNA. Thus, by combining RNAi-mediated knockdown with alternating dietary regimes, *C. elegans* has over the years become an attractive model system for studying gene functions during changing nutritional conditions in particular during dietary restriction (DR). Thus, *C. elegans* has played a crucial role in key discoveries made within aging research.

Aging has largely been defined as a gradual decline of functions at the molecular, cellular, tissue, and organismal level ultimately leading to disease and death [6, 7]. Despite this complexity, the molecular mechanisms governing the aging processes have attracted much attention over the past decades. With the notion that factors modulating lifespan might be the same that influences the process of aging, lifespan has often been monitored simply by measuring the lifetime spanning from birth to death or end of larvae development to death [8]. Klass and coworkers originally identified a class of longevity mutants [9], which later were found to share the same unique genetic locus, which was named *age-1* [10, 11], encoding the catalytic subunit of phosphatidylinositol 3-kinase (PI3K). Soon after, mutations in the insulin/insulin-like growth factor 1 receptor (IGF-1) were found to extend lifespan not only in *C. elegans* [12, 13], but also in rodents and fruit flies [14–16]. These discoveries showed that lifespan is not only orchestrated on a genetic level [8], but also closely linked to metabolic regulation and nutritional cues [17], and thus spurred a powerful entry point for understanding longevity at a molecular level.

In this review, we provide a detailed overview of how lifespan in *C. elegans* is regulated at the molecular level with emphasis on transcriptional and epigenetic regulators. Furthermore, we describe how nutritional and metabolic cues are influencing these specific regulators, especially through dietary restriction. We acknowledge the importance of mitochondria in the regulation of lifespan. However, while mitochondrial regulation of lifespan in *C. elegans* seems to be linked to respiration, generation of radical oxygen species, and mitochondrial fitness, their role in generating substrates for epigenetic modifications of histones in *C. elegans* still remains to be elucidated. We therefore consider this beyond the scope of the present review and kindly encourage readers to consult these reviews for further details [18–21].

### Central nutrient-sensing pathways in lifespan extension

Obesity poses a major risk for serious diet-related diseases, including diabetes mellitus, cardiovascular disease, hypertension and stroke, and certain forms of cancer. Its health consequences range from increased risk of premature death to serious chronic conditions, which reduce the overall quality of life. Oppositely, reduced food intake, also known as caloric, energy, and dietary restriction, comes with several health benefits, which can counteract obesity-induced conditions [22]. In 2009, Greer and Brunet compared different strategies to induce dietary restriction in *C. elegans* [23] and found that different regimes of DR all extend lifespan, however, to different degrees. This was mediated through different nutrient-sensing systems activating different transcription factors, arguing that extension of lifespan is not mediated by a single linear pathway but by multifactorial processes.

The two major nutrient-sensing pathways that have been identified as key modulators of DR-induced longevity are LET-363/mTOR (mechanistic target of rapamycin) and IIS (insulin/insulin-like growth factor 1) signaling. By sensing cellular levels of amino acids and growth factors, the kinase LET-363/mTOR regulates metabolic processes including lysosomal biogenesis, autophagy, and protein and lipid synthesis. In a nutrient-rich state, LET-363/mTOR is located at the lysosomal membrane and is activated by the protein Rheb (Ras homolog enhanced in the brain) [24]. Rheb itself is regulated by the protein complex TSC (tuberous sclerosis 1 and 2), which is the substrate of several kinases that are relaying signals of the cellular metabolic state. When activated, LET-363/mTOR directly phosphorylates and inactivates transcription factors such as DAF-16/FOXO and HLH-30/TFEB [24], rendering them incapable of translocating to the nucleus. Oppositely, under low nutrient levels, the TSC complex inactivates Rheb and thereby LET-363/mTOR, which will dissociate from the lysosomal membrane and thus cannot phosphorylate HLH-30/TFEB and DAF-16/FOXO. Both transcription factors are then able to enter the nucleus and transcribe target genes, including genes encoding protein components that are required for autophagy.

The IIS pathway is likewise modulating longevity and is regulated by changes in nutrient availability. Following normal fed conditions, IIS maintains cell proliferation, protein synthesis, and cell growth. IIS is connected to LET-363/mTOR by several downstream mediator proteins and transcription factors. When activated, the insulin/IGF-1 receptor acts through IRS-1 (insulin receptor substrate 1) that activates PI3K, generating PIP3 (phosphatidylinositol phosphate 3) in the plasma membrane. The increase in PIP3 activates Akt (protein kinase B) that by phosphorylating and inhibiting TSC [25] activates LET-363/mTOR. Under DR, the IIS pathway is not activated and hence does not induce LET-363/mTOR activity, thus promoting lifespan-extending processes.

### HLH-30/TFEB-mediated autophagy is necessary for lifespan extension

Autophagy is a highly evolutionarily conserved cellular degradation process, which under normal conditions maintains a non-toxic environment within most cells, by degrading and recycling misfolded proteins and damaged organelles. However, autophagy has been found to be vital for sustaining metabolic homeostasis when organisms encounter stressful conditions by degrading cellular macromolecules to provide nutrients and molecular building blocks. Autophagy can be induced by several forms of cellular or environmental stress factors, e.g., growth factor deprivation, oxidative stress, and starvation [26]. The process of autophagy is driven by a large conjunction of protein complexes that are tightly coordinated and regulated. Studies in yeast have identified more than 30 autophagy-related proteins (ATGs), many of which have mammalian and nematode orthologues [27]. Autophagy is a multistep process wherein autophagosomes are formed and engulfs targets for degradation. The autophagosome formation is initiated by vesicle nucleation, where an isolation membrane is formed. The isolation membrane is expanded into an autophagosome (vesicle elongation) that can dock and fuse to a lysosome containing lysosomal hydrolases. When fused, the cargo is degraded within the autolysosome and breakdown products are released [28].

**Fig. 1:**
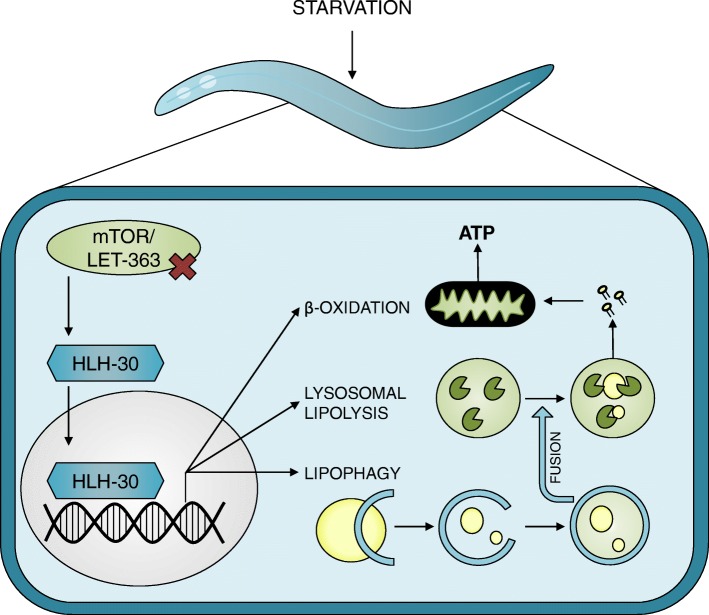
HLH-30/TFEB regulates lipophagy during starvation in *C. elegans*. In response to starvation, the nutrient sensor mTOR/LET-363 is inhibited and the transcription factor HLH-30/TFEB is activated and translocates to the nucleus where it upregulates genes from the CLEAR network. This includes genes that are necessary for all three steps of lipophagy, a selective form of autophagy. In the first step of lipophagy, an autophagosome is formed, engulfing a part of a lipid droplet. In the second step, the sealed autophagosome fuses with a lysosome containing acid lipases that degrades the lipids within the autolysosome. In the final step, free fatty acids are released from the autolysosome and can be utilized for energy production by breakdown through β-oxidation. To date, regulation of β-oxidation has only been shown for TFEB and not for HLH-30 per se

One of the primary regulators of autophagy in metazoans, including *C. elegans*, is the conserved transcription factor HLH-30, an orthologue of the mammalian TFEB (transcription factor EB). HLH-30/TFEB is a member of the basic helix-loop-helix leucine-zipper transcription factor family. HLH-30/TFEB resides as an inactive form in the cytosol under fed conditions. However, once *C. elegans* encounters starvation, HLH-30/TFEB is activated and translocates to the nucleus where it upregulates several groups of genes (Fig. 1) by binding to specific promoter E-box sites transcribing genes from the CLEAR network (Coordinated Lysosomal Expression and Regulation) [29], including those necessary for lysosomal degradation of lipids, a selective form of autophagy known as lipophagy. In this review, we are focusing on the regulation of lipophagy knowing that HLH-30/TFEB activation also regulates other forms of selective autophagies such as mitophagy [30]. Firstly, HLH-30/TFEB upregulates genes necessary for the assembly of the lipophagic machinery, including formation, expansion, and fusion of the autophagosomes that encapsulate lipid droplets. Secondly, expression of genes driving biogenesis of lysosomes is upregulated as well as lysosomal lipases that are required for the degradation of lipids after fusion with autophagosomes [31] (Fig. 1). Lastly, mammalian lipid catabolism genes are upregulated by TFEB, through the activation of the PGC1α-PPARα program, including enzymes for β-oxidation of the fatty acids released from the lysosome [32, 33]. Notably, to date, HLH-30 has not been found to regulate β-oxidation in *C. elegans* per se.

Besides being released from stored triacylglycerols in intestinal lipid droplets by the activity of adipose triglyceride lipase-1 (ATGL-1) in response to starvation [34], fatty acids can also be released by lysosomal engulfment and degradation of lipid droplets. The genome of *C. elegans* comprises eight lysosomal acid lipases (*lipl-1* to *lipl-8*) [35], among which expression of *lipl-1* to *lipl-5* is regulated by HLH-30/TFEB in conjunction with the MaX-like transcription factor MXL-3/MAX. Under fed conditions, MXL-3/MAX suppresses the expression of lysosomal and autophagosomal genes, i.e., *lipl-1* and *lipl-3* [31]. However, upon starvation, MXL-3/MAX is downregulated and allows HLH-30/TFEB to access the promoter region and thus upregulates expression of the lipases that are necessary for the lysosomal breakdown of lipids, ensuring survival during starvation conditions [31, 36]. Hence, the metabolic response controlled by food availability is tightly coordinated, only mobilizing lipids when needed, avoiding an unnecessary and potentially lipotoxic cell environment.

Among the lysosomal lipases, LIPL-4 is particularly interesting as intestinal overexpression of *lipl-4* significantly increases lifespan [37, 38]. Furthermore, LIPL-4 has been found to function interdependently with autophagy in germline-deficient *C. elegans* [39]. Lapierre et al. have shown that the long-lived germline-less *glp-1* mutant has increased levels of autophagy and increased expression of autophagic genes regulated by the transcription factor PHA-4/FOXA. Consistently, they find that levels of LET-363/mTOR are decreased in *glp-1*. Moreover, they show that the upregulation of autophagy is dependent on LIPL-4 activity, which is also increased in *glp-1* animals. Conversely, RNAi of specific autophagic genes significantly reduced the lipase activity of LIPL-4. With this, they provided the first genetic evidence that lipid metabolism and autophagy are linked in modulating longevity in germline-less *C. elegans* [39].

Via its key function in autophagy and lipophagy, HLH-30/TFEB is important for the lifespan extension during starvation [40] and of several long-lived *C. elegans* mutants with increased levels of autophagy [41]. These mutants include *eat-2* (dietary restriction), *daf-2* (impaired insulin signaling), *clk-1* (mitochondrial respiration dysfunction), and *glp-1* (impaired reproduction) [41]. These mutants all comprise genes that collectively affect metabolism in *C. elegans* and henceforth longevity. Oppositely, HLH-30/TFEB extends lifespan when overexpressed further arguing that HLH-30/TFEB functions as a master regulator of autophagy and longevity [41]. Although not found to affect lifespan under normal conditions [40, 41], Lin and colleagues recently found that an *hlh-30* null allele mutant indeed has reduced lifespan under normal conditions but more interestingly promotes stress resistance in cooperation with DAF-16/FOXO [42]. DAF-16/FOXO is well known for its role as a downstream transcription factor of DAF-2/IGF1R in the IIS pathway [13, 43]. By direct interaction, HLH-30/TFEB and DAF-16/FOXO form a transcriptional complex that co-regulates gene expression that promotes survival under oxidative stress resistance [42]. Interestingly, both transcription factors also induce resistance to heat stress, however not via complex formation but through their individual genetic pathway [42]. Furthermore, Lin et al. show that both transcription factors translocate to the nucleus during starvation, indicating that this type of nutritional stress can potentially induce a co-binding transcriptional complex activating gene expression necessary for the survival of starvation.

However, the function of HLH-30/TFEB in longevity is context dependent. While HLH-30/TFEB has mainly been described as an activator of autophagy that induces pro-survival responses under various stress conditions, activation of autophagy by HLH-30/TFEB can surprisingly also have the opposite effect on lifespan. Specifically, lifespan was decreased when worms were fed a high glucose diet, even though HLH-30/TFEB translocates to the nucleus to induce the expression of autophagic genes [44]. This response to high glucose diet has previously been reported, however through different mechanisms. It has been shown that high glucose concentration shortens the lifespan of wildtype worms by downregulating DAF-16/FOXO activity and gene expression of aquaporin, responsible for glycerol transport [45].

The loss of HLH-30/TFEB results in premature death during acute starvation [31, 40], which can be rescued by knockdown of either *vit-1* or *vit-5*, encoding two different vitellogenins [40]. Vitellogenins are precursors of yolk proteins, are crucial for lipid transport to oocytes, and are known to increase with age [46] and to be associated with aging in *C. elegans* [35], thus linking lipoprotein metabolism and transport to starvation survival in *C. elegans* [40].

Interestingly, a recent study has shown a previously unknown and conserved role for HLH-30/TFE B during innate immune response [47]. Post-infection with *Staphylococcus aureus* up to 80% of genes being upregulated in the host response is controlled by HLH-30/TFEB. Genes that are essential for *C. elegans*’ ability to withstand infection included not only antimicrobial but also autophagic genes [47]. Together, these observations indicate that HLH-30/TFEB might be exerting a far broader and more complex regulatory role than previously anticipated. Moreover, these studies underline that not only the activation but also the regulatory functions of HLH-30/TFEB are highly context dependent.

### Additional metabolic regulators of dietary restriction-induced longevity

Besides HLH-30/TFEB other transcription factors are regulating longevity in response to dietary restriction. The transcription factor PHA-4/FOXA is localized to the nucleus under conditions where the activity of LET-363/mTOR is decreased [48, 49]. During dietary restriction, PHA-4/FOXA is responsible for activating the superoxide dismutase genes *sod-1*, *sod-2*, *sod-4*, and *sod-5*, which protect against oxidative stress by removing reactive oxygen species. Furthermore, PHA-4/FOXA is needed for the induction of autophagy in the genetically dietary restricted longevity mutant *eat-2* [48]. Another transcription factor implemented in both oxidative stress resistance and diet-induced longevity is SKN-1/Nrf2. SKN-1/Nrf2 is directly regulated by IIS, and reduced levels of IIS result in the intestinal nuclear accumulation of SKN-1/Nrf2 [50]. When active, SKN-1/Nrf2 upregulates the phase II detoxification system, which is also responsible for detoxifying free oxygen radicals [51, 52]. Moreover, *skn-1* mutants are unable to extend lifespan under bacterial dilution DR showing that SKN-1/Nrf2 is necessary for DR-induced longevity. Interestingly, SKN-1/Nrf2 has more recently been connected to amino acid and lipid metabolism during starvation. It has been shown that mutations in the proline catabolic enzyme *alh-6* increase fat mobilization and fatty acid oxidation in a SKN-1/Nrf2-dependent manner [53].

### Lipid metabolism and lifespan

Lipids are a diverse group of macromolecules, which not only serve as structural components of cellular membranes and as an important energy source, but are also recognized as important bioactive signaling molecules [54]. *C. elegans* does not harbor cells that are dedicated for lipid storage per se as compared to mammalian adipocytes. In *C. elegans*, lipids are primarily stored in the intestine and in skin-like epidermal cells [55]. Furthermore, *C. elegans* is cholesterol auxotroph and does not require cholesterol for membrane integrity but as precursors for signaling molecules [56]. Despite the differences, *C. elegans* provides a powerful model to study lipid metabolism as the majority of lipid metabolic enzymes and pathways are highly evolutionarily conserved (reviewed in [57]). Gao and colleagues recently found that the abundance of most non-esterified FAs is low during development and increases during the reproductive stage, peaking at the post-reproductive stage, while declining during aging [58]. However, the abundance of the very long-chain FAs C24:0, C21:1, and C22:1 peaks at day 10, indicating that these FAs accumulate during the aging process [58]. The phospholipid phosphatidylglycerol and a sphingomyelin species display a similar pattern, being low during the early larval stages while accumulating in late life.

**Fig. 2:**
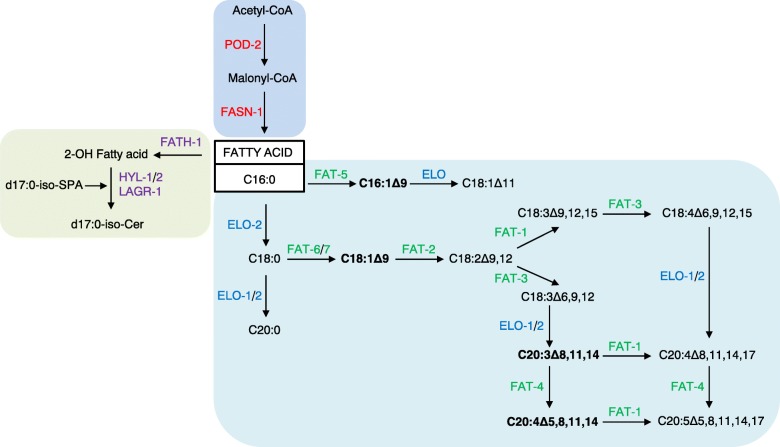
Fatty acid elongation, desaturation, and ceramide synthesis in *C. elegans*. Fatty acid synthesis is orchestrated by the multifunctional enzyme FASN-1 (red). When the fatty acid is synthesized, it can be modified in several ways or enter the synthesis of more complex lipids. Modifications include elongation of chain length by elongases (blue) and introduction of double bonds by desaturases (green). Both classes of enzymes have high specificity towards the fatty acids they modify. Illustrated here is the example of how the fatty acid palmitate (C16:0) can be further modified to monounsaturated and polyunsaturated fatty acids with variating chain length in *C. elegans*. Highlighted in bold are the fatty acids that have been found to be involved in longevity, monounsaturated fatty acids such as C16:1Δ9 and C18:1Δ9 and polyunsaturated fatty acids C20:3Δ8,11,14 (di-homo-γ-linoleic acid, DGLA) and C20:4Δ5,8,11,14 (arachidonic acid, ALA). Furthermore, a simplification of ceramide synthesis is illustrated. The ceramide synthesis is dependent on the enzymes FATH-1, HYL-1/2, and LAGR-1 (purple). Only a selection of fatty acid metabolism is illustrated

Fatty acids are one of the major building blocks used for synthesizing glycero- and phosphoglycero lipids and more complex lipids like ceramides and other sphingolipids. The de novo synthesis of fatty acyl-chains is achieved by the activity of fatty acid synthase, encoded by the *fasn-1* gene, comprising all catalytic activities required for priming, condensation, dehydrogenation, dehydration, and elongation for fatty acid synthesis and termination once the acyl-chain reaches 16 carbons (palmitate). Following termination, fatty acids can be further modified by either elongation or desaturation. In *C. elegans*, elongation is procured by specific elongases encoded by the *elo* genes (*elo-1*, *elo-2*, *elo-5*, and *elo-6*) that are elongating both saturated and unsaturated fatty acids with high specificity [57] (Fig. 2). Introduction of double bonds is carried out by desaturases (*fat-1* to *fat-7*) to produce mono- and polyunsaturated fatty acids [59, 60] (Fig. 2), an important modification that determines the functionality of the fatty acid. When modified, the fatty acids can be incorporated into other major lipids depending on the metabolic state of the cell. For storage, fatty acids are packed as neutral lipids by being esterified with glycerol to form diacylglycerol (DAG), which is further dephosphorylated for the addition of another fatty acid to produce triacylglycerol (TAG) [57]. DAG is a shared intermediate between TAG and phospholipid synthesis. By the addition of different head groups, DAGs can be converted to various phospholipids including phosphatidylcholine and phosphatidylethanolamine which are essential structural lipids incorporated in cellular and organelle membranes.

Fatty acids are also utilized for the synthesis of sphingolipids. The simplest sphingolipid, ceramide, is comprised of a sphinganine base with an attached fatty acid. In *C. elegans*, the sphingoid base is produced from the condensation of serine and branched-chain fatty acid C15:iso to form d17:iso-sphinganine [57]. The addition of the fatty acid to the d17:iso-sphinganine is catalyzed by three ceramide synthases encoded by *hyl-1*, *hyl-2*, and *lagr-1*genes (Fig. 2). HYL-1 and HYL-2 have an affinity for specific fatty acids. HYL-1 transfers distinctly C24–C26 acyl chains whereas HYL-2 transfers C20–C22 acyl chains [61]. Ceramide can be further modified to form more complex sphingolipids such as sphingomyelins and gangliosides making it a central hub for sphingolipid metabolism. Together with phospholipids, sphingolipids preserve cellular membranes; however, these lipids have emerged as important signaling molecules regulating cell growth, senescence, and apoptosis [62] especially sphingosine-1 phosphate and ceramide. Accordingly, RNAi of the ceramide synthase activity motif in *hyl-1* increases the lifespan of *C. elegans* [63] and deletion of both *hyl-1 and lagr-1* increase lifespan compared to wildtype animals. Oppositely, loss of *hyl-2* decreases lifespan. Furthermore, the lifespan extension of *hyl-1;lagr-1* animals depends on not only functional autophagy, but also transcription factors DAF-16/FOXO and SKN-1/Nrf2 [64]. Their differential specificities of the ceramide synthases suggest that particular sphingolipid species are pro-aging, while others support longevity. Thus, loss of HYL-1 and LAGR-1 induces a dietary restriction-like longevity phenotype by upregulating autophagy in an DAF-16/FOXO- and SKN-1/Nrf2-dependent manner possibly induced by changes in the sphingomyelin composition [64].

The insulin receptor mutant *daf-2* and germline-deficient *glp-1* mutant both display an increase in the accumulation of intestinal lipids [13, 65] whereas the dietary restricted *eat-2* mutant has decreased lipid stores [66]. These observations indicate that it might not be the amount of stored lipids themselves that are influencing the lifespan of these mutants. However, it could be that the lipids are used as metabolic signals ensuring lifespan-extending regulation. O’Rourke and colleagues recently provided evidence supporting such a hypothesis, with their study of ω-6 polyunsaturated fatty acids and their involvement in lifespan extension. They show that overexpression of LIPL-4 leads to activation of autophagy through the production of the ω-6 polyunsaturated fatty acids arachidonic acid (AA) and di-homo-γ-linoleic acid (DGLA) and thereby to lifespan extension of *C. elegans* [67]. Furthermore, they propose that AA and DGLA or derivatives hereof also act as signals of low food availability triggering a fasting survival program extending lifespan [67]. Moreover, the fatty acid oleoylethanolamide (OEA) also promotes longevity in response to overexpression of LIPL-4 [38]. OEA binds directly to LBP-8, a lysosomal lipid chaperone that activates nuclear hormone receptors NHR-49/PPAR-α and NHR-80/HNF4 regulating genes involved in β-oxidation and fatty acid desaturation, respectively [35, 68]. Both receptors are known to be necessary for the longevity of several longevity models, including *glp-1* [69]*.* In this way, lysosomal lipolysis is linked to nuclear hormone receptor signaling in promoting longevity in *C. elegans* [38]. Most interestingly, a recent study by Ramachandran and colleagues has uncovered a close relationship between lysosomal lipid signaling and mitochondrial activity in coordinating lipid metabolism, redox homeostasis, and longevity [70]. They show that LIPL-4-LBP-8 signaling increases mitochondrial β-oxidation, reducing lipid storage and promoting longevity in *C. elegans* [70].

It is however definite that lipid accumulation has severe consequences during aging, not just in nematodes but in mammals too, including humans. Ectopic fat accumulation occurs when excess fatty acids are deposited in non-adipose organs or cells. This is often seen in obesity, but it also occurs during aging and increases when an organism reaches high age as cells loose membrane integrity [71]. Age-dependent ectopic fat is deposited specifically in body-wall muscle, neuronal, and pharyngeal cells where the lipid content expands as *C. elegans* ages [72]. This expansion of lipids may lead to lipotoxicity, impairing the cellular function and increasing the progression of age-related diseases [72]. The study of ectopic fat distribution in *C. elegans* by Palikaras et al. revealed a novel role for HLH-30/TFEB in regulating ectopic fat in an autophagy-independent matter in non-stressed wildtype worms. With this, they showed that HLH-30/TFEB is also important for regular lipid metabolism, furthermore suggesting that HLH-30/TFEB could be upholding lipid homeostasis by regulating vitellogenin transport [35, 40].

### Amino acid metabolism and lifespan

Amino acids are crucial building blocks for protein synthesis and act also as key signaling molecules. In *C. elegans*, amino acid concentrations change with age [73] while supplementation of 18 out of 20 individual amino acids extends lifespan [74]. Recently, by investigating the metabolic changes during life history, Gao and colleagues showed that the majority of amino acid species are most abundant during development and decrease during adulthood in *C. elegans* [58]. Oppositely, the abundance of glycine and aspartic acid is lowest during development and early adulthood but increases throughout adulthood and to late age [58]. Accumulation of glycine in aged *C. elegans* is coupled to a decrease in the gene expression of glycine degradation enzymes. Glycine plays an important role in the folate cycle and hence in the synthesis of one-carbon bound tetrahydrofolates (THFs) [75]. THFs are coenzymes in several methylation reactions producing *S*-adenosylmethionine (SAM) through the methionine synthase, SAMS-1, or methionine produced by methionine synthase (METR-1). Dietary supplementation of glycine extends the lifespan of wildtype *C. elegans*, and intriguingly, mutations in *sams-1* and *metr-1* abrogate glycine-dependent lifespan extension, indicating that glycine affects lifespan via the methionine cycle. Accordingly, glycine levels are increased in long-lived *daf-2* and *eat-2* mutants in which glycine, folate-dependent one-carbon, and methionine metabolisms are transcriptionally induced [75].

### Epigenomic changes and lifespan—a new turn in aging research

There is compelling evidence for an epigenetic role in the regulation of lifespan. Epigenetic mechanisms are highly reversible, and therefore, these pathways are closely linked to cell metabolism and nutritional status. Metabolite availability is a determining factor for the modulators of the epigenetic landscape. Dietary restriction is one of the most effective means of extending lifespan; however, the connection between epigenetic regulation and dietary restriction-induced longevity is still unclear. Understanding how dietary restriction leads to metabolic perturbations that modulate epigenetic modifications governing longevity will provide new information on how altering nutritional state can result in a genetic response that potentially delays aging processes. Therefore, it is of great interest to elucidate the link between dietary restriction and the epigenetic events that positively affect lifespan.

The epigenome is comprised of different types of information that in cooperation determines the functions of every cell and fate of organisms. The epigenome comprises chromatin structure remodeling, transcriptional networks, post-translational modifications (PTMs) of histones, DNA methylation, and transcription of non-coding RNAs [76], which all have been found to distinctively influence the aging process, some even to be causative [6].

Chromatin is the polymer of nucleosomes composed of DNA packaging histones. By regulating access of the transcriptional machinery to DNA, chromatin and epigenetic factors regulate gene expression dynamically or even over longer time scales, e.g., through cell division or transgenerations [77]. These factors are enzymes that modify DNA directly or the core histones H2A, H2B, H3, and H4 and some variants [78]. It is the flexible C- and N-terminal tails of these histones that enable transcriptional activation and repression in the form of post-translational modifications. The histone tails can be subjected to a vast group of PTMs that either singularly or in different combinations regulate the accessibility of DNA within the chromatin. Specifically, methylation, acetylation, and phosphorylation represent reversible PTMs that are crucial for the correct chromatin state and thereby gene expression. These PTMs are either removed from or attached to specific amino acid residues (mostly lysine residues) in the histone tails by specific modifying enzymes. By utilizing various metabolites as co-factors, histone methyltransferases (HMTs), histone demethylases (HDMs), histone acyltransferases (HATs), and histone deacetylases (HDACs) are modifying histones to form either heterochromatin or euchromatin and to recruit other regulatory protein complexes and transcription factors. The histone mark patterns define the chromatin state and thereby the level of transcriptional activity of target genes. Therefore, the chromatin structure affects nearly all cellular processes, including those that are linked to aging such as DNA damage repair, impaired DNA replication, and altered transcription [79].

### Chromatin marks and metabolism in lifespan

With age, there is a general loss of histones coupled with local and global chromatin remodeling, an imbalance of activating and repressive histone modifications, and global transcriptional changes [7]. Histone marks and their ability to alter chromatin state are linked to cellular metabolism. The formation of histone marks relies on metabolite availability, either those accessible from cellular pools or those from dietary supplementation. Several metabolites are shared between chromatin remodeling processes and metabolic pathways; examples of these are α-ketoglutarate, *S*-adenosylmethionine (SAM), acetyl coenzyme A (acetyl-CoA), and also lipids themselves [80, 81]. Intriguingly, modification of chromatin enabled by utilizing these metabolites alters the expression of genes involved in regulating lipid metabolism. This reciprocal relationship could indicate that the interaction between the two could be regulating the aging process [80].

Histone acetylation is induced by HATs that utilize acetyl-CoA as a co-factor for the addition of acetyl groups to lysine residues. Acetyl-CoA is the end product of fatty acid breakdown by β-oxidation and a metabolite that is implicated in numerous metabolic processes. The cellular levels of acetyl-CoA and thereby the availability of acetyl groups can therefore modulate the efficiency of the acetylation reaction [81]. The source of acetyl-CoA can be either glucose or acetate depending on the given organism [80]; however, it has recently been shown that a large part of the acetyl groups used for histone acetylation in mammalian cells can be derived from lipids [82]. McDonnell et al. showed that under glucose starvation, up to 90% of the acetyl groups found on histones in cell cultures originate from octanoate [82]. This indicates that the acetyl-CoA needed for acetylation of histones can both depend on the given organism as well as the metabolic state of that organism, determined by nutrient availability. Finally, Eisenberg et al. recently found that high levels of acetate activate the nucleocytosolic acetyl-CoA synthetase Acs2 and subsequent acetyl-CoA-dependent hyperacetylation of histone H2A/H2B and H3 targets and expression of ATG genes in *S. cerevisiae* [83]. Collectively, this suggests that different subcellular pools of acetyl-CoA may contribute differentially to histone modifications and hence regulation of lifespan. Acetylation of histones is associated with heterochromatin formation and active gene expression, e.g., in *C. elegans*, the HAT and CPB-1 are necessary for correct differentiation during embryogenesis by acetylating lysine 5 on histone 4 (H4K5) [79, 84]. It is however so far deacetylation that has mostly been associated with lifespan extension [76, 85].

#### Sirtuins, caloric restriction, and lifespan extension

**Fig. 3:**
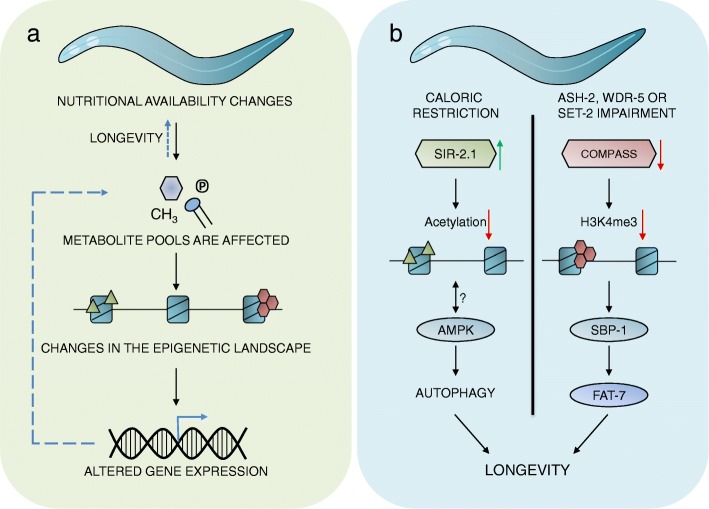
Interconnections between metabolism, epigenetic modifications, and longevity in *C. elegans.* There are tight connections between nutritional status, metabolite availability, and epigenetic modifications that are changing gene expression leading to longevity. **a** When the nutritional status changes, metabolite availability changes too. These changes can affect the post-translational modifications on specific histones and therefore gene expression beneficial for lifespan extension. Altered gene expression can also influence the metabolite pool and induce longevity. **b** Specific examples of what is outlined in **a** Left: Upon caloric restriction, the histone deacetylase SIR-2.1 is upregulated leading to lower levels of acetylation, which has been shown to upregulate autophagy and extend lifespan. Furthermore, sirtuins have been shown to act together with AMPK, a main inducer of autophagy. Therefore, it is possible that the caloric restriction-induced SIR-2.1 activity leads to an increase in AMPK activity, upregulating autophagy resulting in longevity. Right: Impairment of the methyltransferase complex COMPASS in the germline reduces trimethylation of histone 3 lysine 4, which activates the transcription factor SBP-1/SREBP-1 in the intestine. SBP-1/SREBP-1 controls the expression of the fatty acid desaturase FAT-7 that increases the levels of monounsaturated fatty acids leading to longevity. Both examples illustrate how metabolic cues can induce longevity, either through caloric restriction lowering metabolite availability or by reduction of certain histone modifiers leading to increase in specific metabolites

Deacetylation of histones is needed for silencing gene expression, and a specific group of histone NAD-dependent deacetylases, the sirtuins, has been associated with longevity. Deletion or inhibition of sirtuin SIR-2.1 (*C. elegans* orthologue of yeast SIR2 and human SIRT1) reduces lifespan, while increasing the silencing activity of SIR-2.1 extends lifespan [76, 85, 86]. The lifespan extension induced by SIR-2.1 overexpression has been shown to be dependent on the mitochondrial 3-ketoacyl thiolase indicating that fatty acid oxidation is crucial for SIR-2.1-induced longevity [87]. What makes this sirtuin even more interesting in regard to aging is the notion that caloric restriction (CR) induces activation of SIR-2.1/SIRT1 and hence promotes lifespan [76] (Fig. 3). Furthermore, stimulation of SIR-2.1/SIRT1 by CR upregulates autophagy in *C. elegans* and human cells [88]. Moreover, human SIRT1 and AMPK cooperatively induce autophagy by upregulating autophagic genes and by inhibiting mTOR signaling [89]. This shows that it is not only the availability of acetyl-CoA that influences histone acetylation but also the general nutritional state of the organism and that sirtuins play an important role in lifespan extension, perhaps mediated through upregulated autophagy (Fig. 3). This is an interplay that will be important to further investigate, as sirtuins are considered to be great drug targets in promoting longevity and even health span by mimicking CR-induced lifespan extension. Notably, two mitochondrial sirtuins, SIR-2.2 and SIR-2.3, have recently been shown to extend lifespan in a diet-dependent manner when knocked down in *C. elegans* [90]. Furthermore, these isoforms are found to modulate the oxidative stress response, underlining that the function of the sirtuin protein family reaches beyond histone deacetylation.

#### COMPASS, fatty acid desaturation, and lifespan extension

Post-translational methyl histone modifications, such as methylation of lysine residues on histone tails, are another type of epigenetic modification. SAM is a universal donor of methyl groups in methylation reactions in various cellular processes including methylation of histones and lipids. Methylation is important for phospholipid metabolism where SAM is required for the trimethylation of phosphatidylethanolamine (PE) to phosphatidylcholine (PC). Trimethylation is also familiarized with histone modification and especially H3K4 trimethylation (H3K4me3), a transcriptional activating modification, catalyzed by the protein complex COMPASS in *C. elegans* [91]. The COMPASS complex is comprised of several methyltransferases, ASH-2, WDR-5, and SET-2, and depletion of any of these modifiers in the germline has shown to increase the lifespan of adult *C. elegans* [91]. This lifespan extension caused by H3K4me3 modifier deficiency has recently been linked to the enrichment of monounsaturated fatty acids (MUFAs). Evidently, the increase in MUFAs is induced in the absence of H3K4me3, which activates the transcription factor SBP-1/SREBP-1 in the intestine that controls the expression of the fatty acid desaturase FAT-7 [92] (Fig. 3). They furthermore show that dietary supplementation of MUFAs also has a positive effect on lifespan. The exact mechanism by which MUFAs regulate longevity is yet to be resolved but may be linked to changes in membrane fluidity, energy storage, or activation of specific signaling pathways [92]. Intriguingly, it has also been shown that the level of MUFAs relative to PUFAs is increased in long-lived *daf-2* animals in response to DAF-16/FOXO-dependent upregulation of FAT-7 [93].

#### Demethylation, insulin signaling, and longevity

Lifespan can be altered through epigenetic regulation of specific targets in metabolic signaling pathways. The demethylase UTX-1 regulates lifespan by targeting genes in the insulin/IGF-1 signaling pathway in *C. elegans* [94, 95]. UTX-1 is a H3K27 demethylase that by removing this transcriptionally repressive histone mark increases gene expression. Expression of *utx-1* itself increases with age, and RNAi knockdown of *utx-1* extends lifespan by approximately 30% when compared to wildtype worms [94]. UTX-1 targets and regulates among others *daf-2*, the level of which also increases with age, and its downstream targets [94]. Downregulation of *utx-1* extends lifespan in a DAF-16-dependent manner which more frequently translocates to the nucleus upon *utx-1* removal [94]. With these findings, they show that UTX-1 can regulate the H3K27me3 levels on IIS pathway genes, especially *daf-2*, and hence epigenetically regulate gene expression. Via its increase during aging, UTX-1 upregulates IIS, which in turn reduces DAF-16/FOXO levels that compromise cellular maintenance processes and renders the worms less stress resistant and thereby induces an aging-related decline in cellular functions [94].

## Future challenges and conclusion

Aging has intrigued scientists for decades, and the importance of understanding the aging process has only become more evident in recent years. Age-related diseases and especially their onset attract attention as early interventions potentially can ensure healthier aging and perhaps prevent development of certain diseases. *C. elegans* has been at the forefront in discovering that aging is a result of multiple complex molecular mechanisms that are susceptible to genetic and environmental alterations and hence to manipulation by nutrients or by pharmaceuticals. *C. elegans* continues to serve as a highly tractable model system for delineating conserved mechanisms determining for the process of aging, especially in the interest of clarifying the impact of diet-induced metabolic alterations on longevity. That there is a connection between dietary restriction and longevity has been known for a long time and that this connection is rooted in metabolic signaling pathways such as mTOR and IIS, which ultimately regulate key transcription factors that enable cells and organisms to adapt to nutritional changes. However, it has only recently become evident that the transcriptional connection between the two also relies on epigenetic cues. Despite numerous advances in the field, many questions still remain unanswered. Does aging have a beginning? And if so, what age-related event occurs first? What molecular changes are causative to aging and which are simply accompanying aging? Is there one specific epigenetic modification that is the aging determining factor? The challenges in answering these questions lie in the complexity of almost all classes of epigenetic modifications discovered so far are affecting longevity pathways and the fact that still more chromatin marks and gene regulators are being uncovered. It appears that one approach to understanding aging is to delineate key epigenetic mechanisms that specifically affect age-related signaling pathways and how these epigenetic mechanisms are influenced by metabolic status. Moreover, discovering causative epigenetic changes in age-dependent diseases could lead to the identification of specific enzymes that could be therapeutic targets for improving healthspan and extend lifespan. The greatest challenge lies in dissecting the interconnections between specific chromatin-based epigenetic changes and age-related decline in molecular, cellular, and tissue functions leading to disease and death.

## Data Availability

Not applicable
